# Geographic variation in surgery rates among older patients with early (ER positive HER2 negative) breast cancer: Influence of cardiovascular disease and comorbidities: A national registry dataset analysis

**DOI:** 10.1016/j.ejso.2025.110432

**Published:** 2025-12

**Authors:** Emma Crewe, Freya Tyrer, John Deanfield, Mark de Belder, Jennifer Lai, Mamas Mamas, David Adlam, Alistair Ring

**Affiliations:** a Breast Unit – Department of Medicine, The Royal Marsden NHS Foundation Trust UK, and Nuffield Department of Primary Care Heath Sciences, University of Oxford, UK; b Leicester Real World Evidence Unit, Diabetes Research Centre, University of Leicester, UK; c Institute of Cardiovascular Sciences, University College London, London, UK; d National Institute for Cardiovascular Outcomes Research, NHS Arden and Greater East Midlands Commissioning Support Unit, London, UK; e National Disease Registration Service, NHS England, UK; f ACI Audit Lead, School of Medicine, Keele University, UK; g Department of Cardiovascular Sciences, University of Leicester and Leicester National Institute of Health Research Biomedical Research Centre, Glenfield Hospital, Leicester, UK; h The University Hospitals of Leicester NHS Trust, Leicester, UK; i Leicester British Heart Foundation Centre of Research Excellence, UK; j Breast Unit – Department of Medicine, The Royal Marsden NHS Foundation Trust, UK

**Keywords:** Breast cancer, Cardiovascular disease, Older adults, Surgery

## Abstract

**Introduction:**

Women over 70 years of age with operable oestrogen receptor positive (ER positive) breast cancer have worse survival outcomes than younger women. Primary surgery is the optimal treatment with primary endocrine therapy reserved for patients who are unfit or who have multiple co-morbidities. Inferior outcomes in this patient population might be explained by underuse of surgery, the rates of which vary considerably between geographical regions in the UK. We determined the rates of surgery versus primary endocrine therapy in a cohort of women aged over 70 in England, with potentially curable ER positive breast cancer, according to the presence of pre-existing cardiovascular disease (CVD), comorbidities, social deprivation, and by geographical location.

**Materials and methods:**

33,235 women aged 70 years or older with stage I to III ER positive breast cancer from the 20 regional NHS Cancers Alliances in England were identified from the cancer registry. Linked hospital records were used to identify patient demographics, tumour and treatment characteristics, resection rates, CVD prevalence and other co-morbidities.

**Results:**

25,800 (77.6 %) patients underwent surgery, 6787 (20.4 %) patients received primary endocrine therapy alone, 648 (2 %) patients received no treatment. Both CVD and surgery prevalence varied by geographical location. After adjustment for case mix the differences between Cancer Alliances attenuated and no longer reached statistical significance.

**Conclusions:**

We found regional differences in rates of surgery in patients with breast cancer across different centres. After adjustments, the variation is largely attributable to case mix. Under recording of endocrine therapy data in secondary care limits full interpretation.

## 1 Introduction

A third of all diagnoses of breast cancer in the UK are made in women of 70 years of age and over [[1], [2], [3]]. In this age group co-morbidity, including that from cardiovascular disease (CVD), is higher than in younger age groups and may be an important determinant of treatment [1,4]. There are two broad treatment options for older patients with operable hormone receptor positive (ER positive), HER2 negative early breast cancer. These are surgery followed by adjuvant treatment including endocrine therapy, or endocrine therapy alone. Surgery is generally regarded as a more optimal treatment, with several studies showing better survival outcomes in older patients treated with surgery, compared to endocrine therapy, especially when remaining life expectancy exceeds 2–5 years from the point of treatment [1]. Endocrine therapy (which can achieve control for a median of 2–3 years) is suitable for more frail patients who are unfit for surgery and more likely to die from causes other than breast cancer [1]. As a result this more conservative treatment approach might be more appropriate in older adults with pre-existing co-morbidities [1].

Overall in the UK, older women with breast cancer have been shown to have worse outcomes than younger women, with greater rates of non-surgical treatment offered [2]. Previous studies have shown that there is variation in rates of surgery versus endocrine therapy offered to patients over 70 years with operable breast cancer, with regional rates of endocrine therapy use varying between 12 % and 40 % in the UK [2]. It is possible that case mix variations (in cancer stage, age and co-morbidities) explain the difference in rates of surgery. However, it is also important to establish that lower rates of surgery are not associated with other factors, such as social deprivation or treatment centre practice, as there is a risk that this could lead to undertreatment of fitter older patients who will then experience worse outcomes [4].

In this analysis we describe the rates of surgery in a cohort of patients aged 70 years and above with operable breast cancer, according to the presence of pre-existing CVD, social deprivation, and co-morbidities.

## 2 Materials and Methods

This study was reviewed and approved by the Virtual Cardio-Oncology Research Initiative Consortium Project Review Panel. The Virtual Cardio-Oncology Research Initiative research program has received favourable ethical opinion from the Northeast–Newcastle & North Tyneside 2 Research Ethics Committee (reference 18/NE/0123). The study was performed in accordance with the Declaration of Helsinki.

### 2.1 Data sources

This was an observational electronic health record study using data from the Virtual Cardio-Oncology Research Initiative (VICORI) programme [5]. The programme links English cancer registry data from the National Cancer Registration Dataset (NCRD) [6], Hospital Episode Statistics (HES) [7] and cardiovascular audit data from the National Institute for Cardiovascular Outcomes Research (NICOR) [8]. The procedures for linkage are described in detail elsewhere [4,5]. The VICORI Patient and Public Involvement group were formed to set the research agenda for this programme of cardio-oncology research. They were consulted prior to undertaking the study, to support the development of the research questions and updated throughout the delivery of the study and the preparation of this manuscript.

Inclusion/exclusion criteria for this study are shown in Supplementary Table A. In brief, we selected a cohort of female patients aged 70 years and older from NCRD with breast cancer diagnosed from January 1, 2013 to December 1, 2018. Patients were required to have tumours which were suitable for treatment with curative intent (TNM stage I-III) and that were ER positive and HER2 negative. We took the patients’ first diagnosis in the observation window and tumours with the worst prognosis (based on stage, grade and receptor status) where more than one tumour occurred on the same day. Patients with missing tumour stage were excluded.

### 2.2 Outcome and exposure measures

OPCS-4 (Classification of Interventions and Procedures version 4) treatment and procedure codes from NCRD and/or HES, that are used by clinical coders within the NHS, were used to identify resection, Breast surgery was categorized into: breast-conserving surgery (e.g., wide local excision, quadrantectomy), mastectomy (total, skin-sparing, subcutaneous), re-excision, unspecified surgery and no surgery. Supplementary Table B provides full coding details.

CVD was identified from ICD-10 diagnoses recorded in HES admitted patient care (inpatient) data (any diagnostic position; Supplementary Table C) [9] and/or one of the four NICOR audits [8] (Myocardial Ischaemia National Audit Project [MINAP]; National Adult Cardiac Surgery Audit [NACSA]; National Adult Percutaneous Coronary Intervention [NAPCI]; National Heart Failure Audit [NHFA]) within the five years prior to the cancer diagnosis. The following CVD phenotypes were used: cerebrovascular disease (and subgroup stroke); congestive cardiac failure; ischaemic heart disease (and acute myocardial infarction subgroup); peripheral artery disease; and valvular heart disease [4] (Supplementary Table D).

Geographical location was presented using Cancer Alliance derived from hospital postcode. The Cancer Alliances were established by NHS England in 2015 following recommendations by the Independent Cancer Taskforce report; each has an average population of around 2.9 million people. The number of Cancer Alliances and the geographical boundaries change occasionally; the boundaries used here were defined in 2019 [[10], [11], [12]].

### 2.3 Covariates

Demographic information collected comprised age at cancer diagnosis (exact age or grouped into 70–74; 75–79; 80–84; ≥85 years for descriptive statistics), ethnicity (self-assigned, grouped into: white; mixed; Asian black other) and the Income Domain of the Index of Multiple Deprivation (IMD)(2019) quintile (1 = least deprived to 5 = most deprived), the official measure of deprivation in England using patients’ postcodes [13].

Tumour-specific characteristics were: cancer stage (I, II, III; classified using the tumour, nodes, and metastases [TNM] scoring system); histology (ductal, lobular, mixed, other); grade (well differentiated, moderately differentiated, poorly differentiated, undifferentiated/anaplastic, grade inappropriate); screen detected tumour; laterality (left, right, bilateral); progesterone receptor status (positive, negative); and Nottingham prognostic index. Treatment (1 month before, and 12 months after, cancer diagnosis) with chemotherapy and/or radiotherapy was determined using OPCS-4 codes from NCDR/HES. Due to differences in data collection, endocrine therapy within the same timeframe was extrapolated from regimens recorded in Systemic Anti-Cancer Therapy (SACT) or hormone events in NCRD. Binary classification of endocrine therapy receipt (yes/no) was used for consistency. Therapy receipt was counted as "recorded" if endocrine therapy was found in either the SACT or NCRD registry. We did not perform imputation or infer missing data beyond this binary classification. As a result, the true use of endocrine therapy may be higher than represented here, due to under-recording in national datasets.

The Charlson Co-morbidity Index (CCI; 0, 1, 2, 3, ≥4 comorbidity) [14] was used to identify comorbidities within the 5 years before the cancer diagnosis from HES (Admitted Patient Care) data. CVD was removed from the CCI calculation to avoid overlap with the exposure (Supplementary Table E).

### 2.4 Statistical analysis

We explored the relationship between all patient, tumour and treatment characteristics (see covariates) and resection and CVD morbidity using descriptive statistics (proportions and medians; 95 % confidence intervals [CI]; p-values). The relationship between CVD and resection rate was compared using flexible parametric models (5 degrees of freedom), also fitting an interaction between CVD and follow-up time to allow for non-proportional hazards [15]. Age-standardised CVD and resection prevalence by Cancer Alliance, also stratifying by resection type, was also calculated and compared. Funnel plots were used to investigate further any variations in regional resection rates: we calculated the standardised resection ratio for each Cancer Alliance by dividing the observed number of resections in each Cancer Alliance by the predicted number of patient's resections, obtained from a logistic regression model [16] also adjusting for the effects of CVD, age, stage, CCI and IMD. Those Cancer Alliances that fell outside the 99.8 % confidence interval range were flagged as outliers.

All analyses were performed in Stata MP version 17 and R version 4.0.2 [17,18].

## 3 Results

A total of 33,235 patients aged 70 years or older with stage I-III breast cancer and diagnosed with breast cancer between January 1, 2013 to December 1, 2018 were identified. Patient demographics, tumour and treatment characteristics are shown in Table 1. The majority of patients were under the age of 80 years (n = 20,961; 63.1 %) and were White (n = 30,867; 92.9 %). Nearly half (n = 15,617; 47.0 %) had no comorbidities (excluding CVD), as determined using the CCI (Table 1).

**Table 1:** Association between patient, tumour and treatment characteristics and CVD comorbidity.

	CVD comorbidity					Total	
	Yes		No				
	**N**	**(%)**	**N**	**(%)**	**p-value**^a^^a^	**N**	**(%)**
	**5386**	**(100.0) *(16.2 % of total)***	**27,849**	**(100.0) *(83.8 % of total)***		**33,235**	**(100.0)**
**Age category at diagnosis (years), n (% of subgroup; 95 % CI)**							
70–74	1079	(20.0; 19.0, 21.1)	10,880	(39.1; 38.5, 39.6)	<0.001	11,959	(36.0; 35.5, 36.5)
75–79	1314	(24.4; 23.2, 25.5)	7688	(27.6; 27.1, 28.1)	<0.001	9002	(27.1; 26.6, 27.6)
80–84	1318	(24.5; 23.3, 25.6)	5201	(18.7; 18.2, 19.1)	<0.001	6519	(19.6; 19.2, 20.0)
≥85	1675	(31.1; 29.9, 32.3)	4080	(14.7; 14.2, 15.1)	<0.001	5755	(17.3; 16.9, 17.7)
**Ethnicity, n (% of subgroup; 95 % CI)**							
White	5105	(94.8; 94.2, 95.4)	25,762	(92.5; 92.2, 92.8)	<0.001	30,867	(92.9; 92.6, 93.2)
Mixed	12	(0.2; 0.1, 0.3)	53	(0.2; 0.1, 0.2)	0.62	65	(0.2; 0.1, 0.2)
Asian	123	(2.3; 1.9, 2.7)	388	(1.4; 1.3, 1.5)	<0.001	511	(1.5; 1.4, 1.7)
Black	39	(0.7; 0.5, 1.0)	244	(0.9; 0.8, 1.0)	0.27	283	(0.9; 0.8, 1.0)
Other	24	(0.4; 0.3, 0.6)	176	(0.6; 0.5, 0.7)	0.11	200	(0.6; 0.5, 0.7)
*Missing*	*83*	*(1.5; 1.2, 1.9)*	*1226*	*(4.4; 4.2, 4.6)*	*<0.001*	*1309*	*(3.9; 3.7, 4.1)*
**Income domain of the Index of Multiple Deprivation, n (% of subgroup; 95 % CI)**							
1 - Least	1035	(19.2; 18.2, 20.3)	6593	(23.7; 23.2, 24.2)	<0.001	7628	(23.0; 22.5, 23.4)
2	1138	(21.1; 20.0, 25.0)	6834	(24.5; 24.0, 25.0)	<0.001	7972	(24.0; 23.5, 24.4)
3	1149	(21.3; 20.2, 22.8)	5933	(21.3; 20.8, 21.8)	0.96	7082	(21.3; 20.9, 21.7)
4	1055	(19.6; 18.5, 20.6)	4859	(17.4; 17.0, 17.9)	<0.001	5914	(17.8; 17.4, 18.2)
5 - Most	1009	(18.7; 17.7, 19.8)	3630	(13.0; 12.6, 13.4)	<0.001	4639	(14.0; 13.6, 14.3)
**Charlson comorbidity index**^b^**, n (% of subgroup; 95 % CI)**							
0	2546	(47.3; 45.9, 48.6)	13,071	(46.9; 46.3, 47.5)	0.65	15,617	(47.0; 46.5, 47.5)
1	483	(9.0; 8.2, 9.7)	2359	(8.5; 8.1, 8.8)	0.23	2.842	(8.6; 8.3, 8.9)
2	1288	(23.9; 22.8, 25.1)	6868	(24.7; 24.2, 25.2)	0.24	8156	(24.5; 24.1, 25.0)
3	549	(10.2; 9.4, 11.0)	2870	(10.3; 9.9, 10.7)	0.80	3419	(10.3; 10.0, 10.6)
≥4	519	(9.6; 8.8, 10.4)	2445	(8.8; 8.4, 9.1)	0.04	2964	(8.9; 8.6, 9.2)
*Missing*^c^	*1*	*(0; 0, 0.1)*	*236*	*(0.8; 0.7, 1.0)*	*<0.001*	*237*	*(0.7; 0.6, 0.8)*
**TNM stage, n (% of subgroup; 95 % CI)**							
I	2259	(41.9; 40.6, 43.3)	12,507	(44.9; 44.3, 45.5)	<0.001	14,766	(44.4; 43.9, 45.0)
II	2664	(49.5; 48.1, 50.8)	12,795	(45.9; 45.4, 46.5)	<0.001	15,459	(46.5; 46.0, 47.1)
III	463	(8.6; 7.8, 9.3)	2547	(9.1; 8.8, 9.5)	0.20	3010	(9.1; 8.7, 9.4)
**Histology of the tumour, n (% of subgroup; 95 % CI)**							
Ductal	3858	(71.6; 70.4, 72.8)	19,536	(70.1; 69.6, 70.7)	0.03	23,394	(70.4; 69.9, 70.9)
Lobular	876	(16.3; 15.3, 17.3)	5129	(18.4; 18.0, 18.9)	<0.001	6005	(18.1; 17.7, 18.5)
Mixed	98	(1.8; 1.5, 2.2)	705	(2.5; 2.3, 2.7)	0.002	803	(2.4; 2.3, 2.6)
Other	554	(10.3; 9.5, 11.1)	2479	(8.9; 8.6, 9.2)	0.001	3033	(9.1; 8.8, 9.4)
**Grade of the tumour, n (% of subgroup; 95 % CI)**							
Well differentiated	122	(2.3; 1.9, 2.7)	382	(1.4; 1.2, 1.5)	<0.001	504	(1.5; 1.4, 1.6)
Moderately differentiated	931	(17.3; 16.3, 18.3)	4828	(17.3; 16.9, 17.8)	0.93	5759	(17.3; 16.9, 17.7)
Poorly differentiated	3521	(65.4; 64.1, 66.6)	17,915	(64.3; 63.8, 64.9)	0.14	21,436	(64.5; 64.0, 65.0)
Undifferentiated/anaplastic	807	(15.0; 14.0, 15.9)	4699	(16.9; 16.4, 17.3)	0.001	5506	(16.6; 16.2, 17.0)
Grade inappropriate	1	(0.0; 0.0, 0.1)	2	(0.0; 0.0, 0.0)	0.42	3	(0.0; 0.0, 0.0)
*Missing*	*4*	*(0.1; 0.0, 0.1)*	*23*	*(0.1; 0.0, 0.1)*	*0.84*	*27*	*(0.1; 0.1, 0.1)*
**Screen-detected tumour, n (% of subgroup; 95 % CI)**							
Yes	562	(10.4; 9.6, 11.3)	6695	(24.0; 23.5, 24.5)	0.003	7257	(21.8; 21.4, 22.3)
No	3294	(61.2; 59.9, 62.5)	16,436	(59.0; 58.4, 59.6)	<0.001	19,730	(59.4; 58.8, 59.9)
*Missing*	*1530*	*(28.4; 27.2, 29.6)*	*4718*	*(16.9; 16.5, 17.4)*	*<0.001*	*6248*	*(18.8; 18.4, 19.2)*
**Laterality, n (% of subgroup; 95 % CI)**							
Left	2749	(51.0; 49.7, 52.4)	14,271	(51.2; 50.7, 51.8)	0.78	17,020	(51.2; 50.7, 51.7)
Right	2537	(47.1; 45.8, 48.4)	13,081	(47.0; 46.4, 47.6)	0.86	15,618	(47.0; 46.5, 47.5)
Bilateral	98	(1.8; 1.5, 2.2)	492	(1.8; 1.6, 1.9)	0.79	590	(1.8; 1.6, 1.9)
Missing	*2*	*(0.0; 0.0, 0.1)*	*5*	*(0.0; 0.0, 0.0)*	*0.38*	*7*	*(0.0; 0.0, 0.0)*
**Progesterone receptor status, n (% of subgroup; 95 % CI)**							
Positive	2646	(49.1; 47.8, 50.5)	13,044	(46.8; 46.3, 47.4)	0.002	15,690	(47.2; 46.7, 47.7)
Negative	461	(8.6; 7.8, 9.3)	2362	(8.5; 8.2, 8.8)	0.85	2823	(8.5; 8.2, 8.8)
Missing	*2279*	*(42.3; 41.0, 43.6)*	*12,443*	*(44.7; 44.1, 45.3)*	*0.001*	*14,722*	*(44.3; 43.8, 44.8)*
**Nottingham prognostic index, n**	**2619**	**(48.6)**	**21,267**	**(76.4)**	**-**	**23,886**	**(71.9)**
**median (95 % CI)**	3.52	(3.50, 3.56)	3.48	(3.48, 3.50)	0.01	3.50	(3.48, 3.50)
**Surgery, n (% of subgroup; 95 % CI)**							
Yes	2872	(53.3; 52.0, 54.9)	22,928	(82.3; 81.9, 82.8)	<0.001	25,800	(77.6; 77.2, 78.1)
No	2514	(46.7; 45.3, 48.0)	4921	(17.7; 17.2, 18.1)	<0.001	7435	(22.4; 21.9, 22.8)
**Type of surgery, n (% of subgroup; 95 % CI)**							
Breast conserving	1666	(58.0; 56.2, 59.8)	14,379	(62.7; 62.1, 63.3)	<0.001	16,045	(62.2; 61.6, 62.8)
Mastectomy	889	(31.0; 29.3, 32.7)	6075	(26.5; 26.0, 27.1)	<0.001	6964	(27.0; 26.5, 27.5)
Re-excision	4	(0.1; 0.0, 0.3)	92	(0.4; 0.3, 0.5)	0.03	96	(0.4; 0.3, 0.5)
Unspecified breast surgery	313	(10.9; 9.8, 12.1)	2382	(10.4; 10.0, 10.8)	0.40	2695	(10.4; 10.1, 10.8)
**Radiotherapy**^d^**, n (% of subgroup; 95 % CI)**							
Yes	1728	(32.1; 30.8, 33.3)	15,779	(56.7; 56.1, 57.2)	<0.001	17,507	(52.7; 52.1, 53.2)
No	3658	(67.9; 66.7, 69.2)	12,070	(43.3; 42.8, 43.9)	<0.001	15,728	(47.3; 46.8, 47.9)
**Chemotherapy**^d^**, n (% of subgroup; 95 % CI)**							
Yes	157	(2.9; 2.5, 3.4)	2176	(7.8; 7.5, 8.1)	<0.001	2333	(7.0; 6.7, 7.3)
No	5229	(97.1; 96.6, 97.5)	25,676	(92.2; 91.9, 92.5)	<0.001	30,905	(93.0; 92.7, 93.3)
**Endocrine therapy**^e^**, n (% of subgroup; 95 % CI)**							
Yes	4074	(75.6; 74.5, 76.8)	17,412	(62.5; 62.0, 63.1)	<0.001	21,486	(64.6; 64.1, 65.2)
No	1312	(24.4; 23.2, 25.5)	10,437	(37.5; 36.9, 38.0)	<0.001	11,749	(35.4; 34.8, 35.9)

Abbreviations: CVD: cardiovascular disease; CI: confidence interval.

a Chi square or non-parametric equality of medians (continuous).

b Five years before diagnosis, excluding cardiovascular disease.

c Missing if no linkage with Hospital Episode Statistics.

d Identified using National Cancer Registration and Analysis Service treatment standard operating procedure (between 1 month before and 12 months after cancer diagnosis).

e Endocrine therapies not consistently collected by NCRAS. Identified from regimen's recorded in SACT or hormone event in NCRAS (between 1 month before and 12 months after cancer diagnosis).

A total of 5386 (16.2 %) had pre-existing CVD comorbidity, most commonly ischaemic heart disease (n = 3093; 9.3 %) followed by congestive cardiac failure (n = 1397; 4.2 %), cerebrovascular disease (n = 1396; 4.2 %), valvular heart disease (n = 1114; 3.4 %) and peripheral artery disease (n = 665; 2.0 %) (Table 2)**.** On average patients had two or more CVD conditions over the 5-year period.

**Table 2:** Association between patient, tumour and treatment characteristics and surgery.

	Surgery^a^					Total	
	Yes		No				
	**N**	**(%)**	**N**	**(%)**	**p-value**^b^		
	**25,800**	**(100.0) *(77.6 % of total)***	**7435**	**(100.0) *(22.4 % of total)***		**33,235**	**(100.0)**
**CVD comorbidity, n (% of subgroup; 95 % CI)**							
Yes	2872	(11.1; 10.7, 11.5)	2514	(33.8; 32.7, 34.9)	<0.001	5386	(16.2; 15.8, 16.6)
No	22,928	(88.9; 88.5, 89.3)	4921	(66.2; 65.1, 67.3)	*<0.001*	27,849	(83.8; 83.4, 84.2)
**CVD category**^c^**(identified in HES only), n (% of subgroup; 95 % CI)**							
Cerebrovascular	519	(2.0; 1.8, 2.2)	877	(11.8; 11.1, 12.5)	<0.001	1396	(4.2; 4.0, 4.4)
*Stroke*	*221*	*(0.9; 0.7, 1.0)*	*362*	*(4.9; 4.4, 5.4)*	*<0.001*	*583*	*(1.8; 1.6, 1.9)*
Congestive cardiac failure	502	(1.9; 1.8, 2.1)	895	(12.0; 11.3, 12.8)	<0.001	1397	(4.2; 4.0, 4.4)
Ischaemic heart disease	1796	(7.0; 6.7, 7.3)	1297	(17.4; 16.6, 18.3)	<0.001	3093	(9.3; 9.0, 9.6)
*Acute myocardial infarction*	*1768*	*(6.9; 6.5, 7.2)*	*1260*	*(16.9; 16.1, 17.8)*	*<0.001*	*3028*	*(9.1; 8.8, 9.4)*
Peripheral artery disease	361	(1.4; 1.3, 1.5)	304	(4.1; 3.6, 4.5)	*<0.001*	665	(2.0; 1.9, 2.2)
Valvular heart disease	543	(2.1; 1.9, 2.3)	571	(7.7; 7.1, 8.3)	<0.001	1114	(3.4; 3.2, 3.5)
**Age category at diagnosis (years), n (% of subgroup; 95 % CI)**							
70–74	11,233	(43.5; 42.9, 44.1)	726	(9.8; 9.1, 10.4)	<0.001	11,959	(36.0; 35.5, 36.5)
75–79	7905	(30.6; 30.1, 31.2)	1097	(14.8; 13.9, 15.6)	<0.001	9002	(27.1; 26.6, 27.6)
80–84	4606	(17.9; 17.4, 18.3)	1913	(25.7; 24.7, 26.7)	<0.001	6519	(19.6; 19.2, 20.0)
≥85	2056	(8.0; 7.6, 8.3)	3699	(49.8; 48.6, 50.9)	<0.001	5755	(17.3; 16.9, 17.7)
**Ethnicity, n (% of subgroup; 95 % CI)**							
White	24,025	(93.1; 92.8, 93.8)	6842	(92.0; 91.4, 92.6)	0.001	30,867	(92.9; 92.6, 93.2)
Mixed	45	(0.2; 0.1, 0.2)	20	(0.3; 0.2, 0.4)	0.10	65	(0.2; 0.1, 0.2)
Asian	394	(1.5; 1.4, 1.7)	117	(1.6; 1.3, 1.9)	0.77	511	(1.5; 1.4, 1.7)
Black	230	(0.9; 0.8, 1.0)	53	(0.7; 0.5, 0.9)	0.14	283	(0.9; 0.8, 1.0)
Other	161	(0.6; 0.5, 0.7)	39	(0.5; 0.4, 0.7)	0.33	200	(0.6; 0.5, 0.7)
*Missing*	*945*	*(3.7; 3.4, 3.9)*	*364*	*(4.9; 4.4, 5.4)*	*<0.001*	*1309*	*(3.9; 3.7, 4.1)*
**Income domain of the Index of Multiple Deprivation, n (% of subgroup; 95 % CI)**							
1 - Least	6207	(24.1; 23.5, 24.6)	1421	(19.1; 18.2, 20.0)	*<0.001*	7628	(23.0; 22.5, 23.4)
2	6443	(25.0; 24.4, 25.5)	1529	(20.6; 19.6, 21.5)	*<0.001*	7972	(24.0; 23.5, 24.4)
3	5457	(21.2; 20.7, 21.6)	1625	(21.9; 20.9, 22.8)	0.19	7082	(21.3; 20.9, 21.7)
4	4400	(17.1; 16.6, 17.5)	1514	(20.4; 19.4, 21.3)	*<0.001*	5914	(17.8; 17.4, 18.2)
5 - Most	3293	(12.8; 12.4, 13.2)	1346	(18.1; 17.2, 19.0)	*<0.001*	4639	(14.0; 13.6, 14.3)
**Charlson comorbidity index**^d^**, n (% of subgroup; 95 % CI)**							
0	12,248	(47.5; 46.9, 48.1)	3369	(45.3; 44.2, 46.4)	<0.001	15,617	(47.0; 46.5, 47.5)
1	2200	(8.5; 8.2, 8.9)	642	(8.6; 8.0, 9.3)	0.77	2.842	(8.6; 8.3, 8.9)
2	6312	(24.5; 23.9, 25.0)	1844	(24.8; 23.8, 25.8)	0.55	8156	(24.5; 24.1, 25.0)
3	2665	(10.3; 10.0, 10.7)	754	(10.1; 9.5, 10.8)	0.64	3419	(10.3; 10.0, 10.6)
≥4	2305	(8.9; 8.6, 9.3)	659	(8.9; 8.2, 9.5)	0.85	2964	(8.9; 8.6, 9.2)
*Missing*^e^	*70*	*(0.3; 0.2, 0.3)*	*167*	*(2.2; 1.9, 2.6)*	*<0.001*	*237*	*(0.7; 0.6, 0.8)*
**TNM stage, n (% of subgroup; 95 % CI)**							
I	12,166	(47.2; 46.5, 47.8)	2600	(35.0; 33.9, 36.1)	<0.001	14,766	(44.4; 43.9, 45.0)
II	11,380	(44.1; 43.5, 44.7)	4079	(54.9; 53.7, 56.0)	<0.001	15,459	(46.5; 46.0, 47.1)
III	2254	(8.7; 8.4, 9.1)	756	(10.2; 9.5, 10.9)	<0.001	3010	(9.1; 8.7, 9.4)
**Histology of the tumour, n (% of subgroup; 95 % CI)**							
Ductal	18,104	(70.2; 69.6, 70.9)	5290	(71.1; 70.1, 72.2)	0.10	23,394	(70.4; 69.9, 70.9)
Lobular	4750	(18.4; 17.9, 18.9)	1255	(16.9; 16.0, 17.7)	0.002	6005	(18.1; 17.7, 18.5)
Mixed	690	(2.7; 2.5, 2.9)	113	(1.5; 1.2, 1.8)	<0.001	803	(2.4; 2.3, 2.6)
Other	2256	(8.7; 8.4, 9.1)	777	(10.5; 9.8, 11.1)	<0.001	3033	(9.1; 8.8, 9.4)
**Grade of the tumour, n (% of subgroup; 95 % CI)**							
Well differentiated	158	(0.6; 0.5, 1.7)	346	(4.7; 4.2, 5.1)	<0.001	504	(1.5; 1.4, 1.6)
Moderately differentiated	4495	(17.4; 17.0, 17.9)	1264	(17.0; 16.1, 17.9)	0.40	5759	(17.3; 16.9, 17.7)
Poorly differentiated	16,527	(64.1; 63.5, 64.6)	4909	(66.0; 64.9, 67.1)	0.002	21,436	(64.5; 64.0, 65.0)
Undifferentiated/anaplastic	4596	(17.8; 17.3, 18.3)	910	(12.2; 11.5, 13.0)	<0.001	5506	(16.6; 16.2, 17.0)
Grade inappropriate	2	(0.0; 0.0, 0.0)	1	(0.0; 0.0, 0.0)	0.65	3	(0.0; 0.0, 0.0)
*Missing*	*22*	*(0.1; 0.0, 0.1)*	*5*	*(0.1; 0.0, 0.1)*	*0.63*	*27*	*(0.1; 0.1, 0.1)*
**Screen detected tumour, n (% of subgroup; 95 % CI)**							
Yes	7004	(27.1; 26.6, 27.7)	253	(3.4; 3.0, 3.8)	<0.001	7257	(21.8; 21.4, 22.3)
No	15,633	(60.6; 60.0, 61.2)	4097	(55.1; 54.0, 56.2)	<0.001	19,730	(59.4; 58.8, 59.9)
*Missing*	*3163*	*(12.3; 11.9, 12.7)*	*3085*	*(41.5; 40.4, 42.6)*	*<0.001*	*6248*	*(18.8; 18.4, 19.2)*
**Laterality, n (% of subgroup; 95 % CI)**							
Left	13,218	(51.2; 50.6, 51.8)	3802	(51.1; 50.0, 52.3)	0.88	17,020	(51.2; 50.7, 51.7)
Right	12,170	(47.2; 46.6, 47.8)	3448	(46.4; 45.2, 47.5)	0.23	15,618	(47.0; 46.5, 47.5)
Bilateral	407	(1.6; 1.4, 1.9)	183	(2.5; 2.1, 2.8)	<0.001	590	(1.8; 1.6, 1.9)
*Missing*	*5*	*(0.0; 0.0, 0.0)*	*2*	*(0.0; 0.0, 0.1)*	*0.69*	*7*	*(0.0; 0.0, 0.0)*
**Progesterone receptor status, n (% of subgroup; 95 % CI)**							
Positive	12,055	(46.7; 46.1, 47.3)	3635	(48.9; 47.8, 50.0)	0.001	15,690	(47.2; 46.7, 47.7)
Negative	2233	(8.7; 8.3, 9.0)	590	(7.9; 7.3, 8.6)	0.05	2823	(8.5; 8.2, 8.8)
*Missing*	*11,512*	*(44.6; 44.0, 44.8)*	*3210*	*(43.2; 42.0, 44.3)*	*0.03*	*14,722*	*(44.3; 43.8, 44.8)*
**Nottingham prognostic index, n**	**23,189**	**(89.9)**	**697**	**(9.4)**		**23,886**	**(71.9)**
**median (95 % CI)**	3.50	(3.48, 3.50)	3.50	(3.46, 3.60)	0.38	3.50	(3.48, 3.50)
**Radiotherapy, n (% of subgroup; 95 % CI)**							
Yes	17,132	(66.4; 65.8, 67.0)	375	(5.0; 4.5, 5.5)	<0.001	17,507	(52.7; 52.1, 53.2)
No	8668	(33.6; 33.0, 34.2)	7060	(95.0; 94.5, 95.5)	<0.001	15,728	(47.3; 46.8, 47.9)
**Chemotherapy, n (% of subgroup; 95 % CI)**							
Yes	2143	(8.3; 8.0, 8.6)	187	(2.5; 2.2, 2.9)	<0.001	2330	(7.0; 6.7, 7.3)
No	23,657	(91.7; 91.4, 92.0)	7248	(97.5; 97.1, 97.8)	<0.001	30,905	(93.0; 92.7, 93.3)
**Endocrine therapy**^f^**, n (% of subgroup; 95 % CI)**							
Yes	14,699	(57.0; 56.4, 57.6)	6787	(91.3; 90.6, 91.9)	<0.001	21,486	(64.6; 64.1, 65.2)
No	11,101	(43.0; 42.4, 43.6)	648	(8.7; 8.1, 9.4)	<0.001	11,749	(35.4; 34.8, 35.9)

Abbreviations: CVD: cardiovascular disease; CI: confidence interval.

a Identified using National Cancer Registration and Analysis Service treatment standard operating procedure (between 1 month before and 12 months after cancer diagnosis).

b Chi square or non-parametric equality of medians (continuous).

c One person could have more than one CVD event.

d Five years before diagnosis, excluding cardiovascular disease.

e Missing if not linked to Hospital Episode Statistics.

f Identified from regimen's recorded in SACT or hormone event in NCRAS (between 1 month before and 12 months after cancer diagnosis).

Compared to those without CVD, patients with CVD were more likely to be older (≥85yrs: 31.1 % vs 14.7 %; p < 0.001) and to have a higher index of multiple deprivation (18.7 % vs 13.0 %, in IMD 5, most deprived, p < 0.001). Conversely, patients with CVD were less likely to have screen-detected tumours (10.4 % vs 24.0 %; p < 0.003, where data available), less likely to undergo surgery (53.3 % vs 82.3 %; p < 0.001), radiotherapy (32.1 % vs 56.7 %; p < 0.001) or chemotherapy (2.9 % vs 7.8 %; p < 0.001) and more likely to receive endocrine therapy (75.6 % vs 62.5 %; p < 0.001) (Table 1).

Over three-quarters of the sample (n = 25,800; 77.6 %) underwent surgery (Table 2), most commonly breast conserving surgery (n = 16,045; 62.1 %) (Table 1). Those who underwent surgery were more likely to be younger, with 93.9 % of the total patients in the aged 70–74 years subcategory undergoing surgery, compared with 35.7 % of those in the aged 85 years or older subcategory (p < 0.001)(Table 2). Patients with screen detected tumours were more likely to have surgery than those whose breast cancer was detected via a non-screening route (96.5 % of the total number of patients with screen detected tumours received surgery vs 79.2 % of those detected via non screening), p < 0.001)(Table 2), as were those with earlier stage cancers: 82.3 %, 73.6 %, 74.9 %, stages I-III (p < 0.001).

**Fig. 1A–B:**
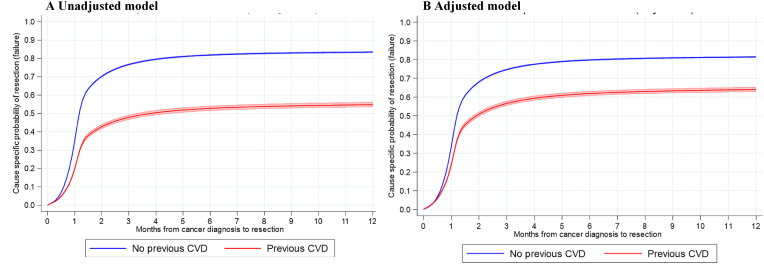
Flexible parametric model showing probability of breast surgery after cancer diagnosis N = 32,998∗. ∗Adjustments made for age, stage, IMD and CCI. Patients with missing CCI excluded. *Abbreviations: CVD = Cardiovascular Disease*.

Fig. 1A and B shows the unadjusted and adjusted probability of surgery over time, according to presence of CVD.

**Fig. 2:**
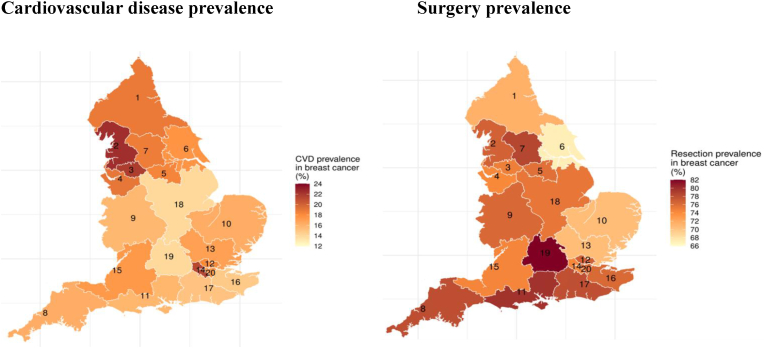
Key Findings: geographical variation in [age-standardised] resection and CVD prevalence in breast cancer patients (N = 33,235). 1 - North East and Cumbria; 2 - Lancashire and South Cumbria; 3 - Greater Manchester; 4 - Cheshire and Merseyside; 5 - South Yorkshire and Bassetlaw; 6 - Humber, Coast and Vale; 7 - West Yorkshire and Harrogate; 8 – Peninsula; 9 - West Midlands; 10 - East of England - North; 11 - Wessex; 12 - North Central and North East London; 13 - East of England – South; 14 - North West and South West London; 15 – Somerset, Wiltshire, Avon and Gloucestershire; 16 - Kent and Medway; 17 - Surrey and Sussex; 18 - East Midlands; 19 - Thames Valley; 20 - South East London. Abbreviations: CVD = Cardiovascular Disease.

After adjustment for age, stage, IMD and CCI, differences between people with and without CVD attenuated, but remained. Both CVD and surgery prevalence varied by geographical location, with CVD rates ranging from 14.0 % (East Midlands) to 22.4 % (Lancashire and South Cumbria) and surgery rates from 67.2 % (Humber, Coast and Vale) to 82.0 % (Thames Valley) (Fig. 2.). Of the types of surgery, rates of breast-conserving surgery were lowest in the North East and Cumbria (41.0 %) and highest in South Yorkshire and Bassetlaw (55.7 %). Rates of mastectomy were lowest in Kent and Medway (16.4 %) and highest in Lancashire and South Cumbria (29.8 %).

As described above we used funnel plots to investigate which characteristics explained variations in regional resection rates. We adjusted for CVD comorbidity to understand if CVD comorbidity explained resection variation. Logistic models were then progressively adjusted for main effects of age at diagnosis, cancer stage, income domain of IMD and CCI (excluding CVD comorbidities).

**Fig. 3:**
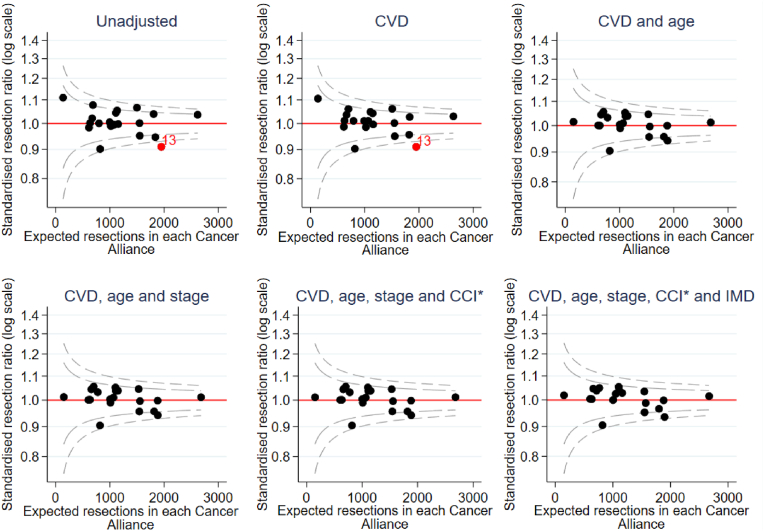
Standardised resection ratio for each Cancer Alliance, adjusting for variables listed in the title of each graph (N = 30,433). ∗ Excluding patients with missing Charlson comorbidity index. *Abbreviations: CVD – Cardiovascular Disease, CCI = Charlson comorbidity index, IMD= Index of Multiple Deprivation*.

The unadjusted and CVD-adjusted funnel plot revealed lower rates of surgery in East of England – South (labelled ‘13’ in Fig. 3) (Fig. 3) than expected across the groups. However, once adjusted for age, the differences between Cancer Alliances attenuated and no longer reached statistical significance.

## 4 Discussion

This analysis was a large scale, population-based analysis describing CVD prevalence in older women with potentially curable breast cancer. Understanding the relationship between breast cancer, comorbidities and CVD is important when planning treatment, analysing outcomes, and developing health care strategies [4].

We used linked national datasets of patients diagnosed with potentially curable breast cancer over 6 years in England and found an overlap between breast cancer and CVD in 16.2 % of patients, which is consistent with rates of CVD co-morbidity in cancer patients generally, as highlighted in a previous analysis [4]. Overall, 52.3 % of patients in this analysis had >1 co-morbidity (excluding CVD), which is less than that reported in a previous analysis of English National Cancer Diagnosis Audit data linked to primary care records which showed that 75 % of women across all tumour types and age groups had at least one pre-existing comorbidity (including CVD) with prevalence increasing greatly with age. [4,19].

While case mix factors such as age and tumour stage explained much regional surgical variation, pre-existing cardiovascular disease remained independently associated with lower surgery rates even after adjustment.

These findings highlight the need for nuanced, individualized surgical decision-making in older breast cancer patients, ensuring that comorbidities such as CVD are assessed rigorously, but do not result in automatic exclusion from surgery when benefits outweigh risks.

Multiple prior studies have demonstrated significant variation in surgical treatment of older patients, often attributed to regional clinical practice norms, surgical decision making, the use of frailty assessments based solely on fitness or performance status and use of multi-disciplinary validated patient assessments, such as the Comprehensive Geriatric Assessment, which when optimised could help to pre-habilitate seemly frailer patients, so that they are fit enough for surgery [1,20,21].

Patient-led decision could also play a role, as some older patients might place a higher value on quality of life rather than quantity, and wish to retain their independence, which may be more preserved with endocrine therapy [1,21].

Our findings add to this evidence, emphasizing that patient-level comorbidities account for most observed variation [3].

### 4.1 Study limitations

Although the older age of patients might be expected to lead to higher rates of CVD and co-morbidity, the somewhat lower frequency compared to other tumour types is likely due to sex differences and breast cancer not sharing risk factors for CVD to the same extent as other malignancies [4]. Further to this, our analysis focused on hospitalisations and did not investigate primary care CVD burden. Due to potential gaps between primary and secondary care patient records, the prevalence of CVD may have been underestimated, as it will have missed CVD diagnosed and managed by primary care in the community.

Due to known limitations in national datasets regarding endocrine therapy documentation, data from SACT and NCRD were cross-referenced. However, Primary Care records provide a much more reliable source for recording ET prescriptions than secondary care alone. Secondary Care hospital records often fail to capture first ET prescriptions, especially in certain subgroups. Linking Primary Care Prescription Database (PCPD) with Secondary Care Data (SCD) could have created a more reliable dataset. Our dataset may underestimate actual ET use, consistent with prior findings [22]. Further to this, the possibility of regional differences in data completeness affecting apparent variation in endocrine therapy or surgical rates.

This analysis did not assess geographic variations in rates of surgery or treatment type by race, nor did it report on variation in rates of CVD by race, both of which are interesting areas for future study.

The National Cancer Registration and Analysis Service (NCRAS) is a clinically rich data resource that registers all patients diagnosed with cancer in England, including information on diagnosis, treatment, and outcomes [23]. Individuals can request to be removed from the NCRAS dataset; however, very few choose to opt out entirely from the cancer registry (<1 in 10,000), and the resulting effect is likely to be minimal [23]. We acknowledge, however, that our cohort may still represent a subset of the national cancer patient population in England, with the possibility of incomplete case ascertainment. Some patients may not have been recorded—such as those who are frail, at the end of life, or not referred to cancer services—which may limit generalizability. Our findings should therefore be interpreted in this context.

## 5 Conclusion

Co-existent CVD and other co-morbidities may affect treatment decisions in early breast cancer in two ways. In the context of high risk of death from competing causes of mortality a more conservative approach may be taken as the early breast cancer will not be a determinant of longevity. Secondly, the presence of pre-existing CVD, and congestive heart failure in particular, might be seen as a contraindication to pursuing surgery and other types of anti-cancer treatments.

This analysis demonstrates geographic variation in CVD rates. This variation could impact overall survival but also cancer specific survival by influencing anticancer treatments offered and outcomes regionally [4]. There were also differences in rates of surgery, as have been previously reported [2]. However, the funnel plots demonstrate that when rates of surgery are adjusted for factors which might put patients at higher risk of death from other causes or treatment related complications (such as age, CVD, other co-morbidities) or need for more extensive surgery (stage), there were no longer significant differences between surgical rates between Cancer Alliances. These findings suggest that the different rates of surgery between different centres are largely observed as a result of case mix. Even after adjustment there remains some variation in surgery rates between regions, which is unexplained. Other (unmeasured) factors, including regional clinical practice and differences in patient preferences in different areas could play a part. These findings underscore the importance of standardized assessment tools and shared decision-making to ensure equitable treatment access.

## Credit author statement

EC and AR designed, wrote and revised the manuscript. DA designed the study and secured funding. AR and DA co-supervised the project. FT analysed the data and wrote the statistical analysis subsection. All authors participated in reviewing the manuscript. AR and DA approved the final version of the manuscript. AR is the guarantor.

## Disclosures

^a^Dr Crewe receives funding from the 10.13039/501100000769University of Oxford for doctoral research in breast cancer unrelated to this report; has received a travel grant from the Patricia McGregor Fund; and has received travel expenses from 10.13039/100004319Pfizer and 10.13039/100005564Gilead.

^b^Dr Tyrer has nil relevant to disclose.

^c^Prof. Deanfield has received grants from British Heart Foundation and Alzheimer's Research UK; receives consulting fee from Amgen, Boehringer Ingelheim, AstraZeneca, Merck, Pfizer, Aegerion, Novartis, Sanofi, Takeda, Novo Nordisk, and Bayer; and Honoraria from Amgen,

Boehringer Ingelheim, Merck, Pfizer, Aegerion, Novartis, Sanofi, Takeda, Novo Nordisk, and Bayer.

^d^Prof. de Belder has nil relevant to disclose

^e^Dr Lee has nil relevant to disclose.

^f^Prof. Mamas has nil relevant to disclose.

^g^Prof. Adlam has received grants from NIHR UK and Heart Research UK for unrelated research; has received an educational grant from Abbott Vascular to support a clinical research fellow for unrelated work; receives royalties from Elsevier for ECG made Practical and ECG Problems; has a patent filed for a Cardiac assist device and patent pending for a cardiac shunt device; is on the advisory board for BEATSCAD, the data and safety monitoring board for PHOENIX; and is chair of ESC-ACVC SCAD Study Group.

^h^Dr Ring has received advisory board fees from Novartis, AstraZeneca, Daiichi Sankyo, Gilead, Merck Sharpe & Dohme; speaker fees from Pfizer, Eli Lilly and Zuellig Pharma; and travel expenses from Roche.

## Funding

This study was funded by a joint research grant from the 10.13039/501100000274British Heart Foundation (SP/16/5/32415) and 10.13039/501100000289Cancer Research UK (C53325/A21134). The funders did not have any involvement in producing the report.

## Declaration of interests statement

EC receives funding from the 10.13039/501100000769University of Oxford for doctoral research unrelated to this report; has received a travel grant from the Patricia McGregor Fund; and has received travel expenses from 10.13039/100004319Pfizer and 10.13039/100005564Gilead.

AR has received advisory board fees from 10.13039/100004336Novartis, 10.13039/100004325AstraZeneca, 10.13039/501100022274Daiichi Sankyo, 10.13039/100005564Gilead, 10.13039/100004334Merck Sharpe & Dohme; speaker fees from 10.13039/100004319Pfizer, Eli Lilly and Zuellig Pharma; and travel expenses from 10.13039/100004337Roche.

DA has received grants from 10.13039/501100000272NIHR UK and 10.13039/501100000327Heart Research UK for unrelated research; has received an educational grant from 10.13039/100011949Abbott Vascular to support a clinical research fellow for unrelated work; receives royalties from Elsevier for *ECG made Practical* and *ECG Problems*; has a patent filed for a Cardiac assist device and patent pending for a cardiac shunt device; is on the advisory board for BEATSCAD, the data and safety monitoring board for PHOENIX; and is chair of ESC-ACVC SCAD Study Group.

JD has received grants from 10.13039/501100000274British Heart Foundation and Alzheimer's Research UK; receives consulting fee from 10.13039/100002429Amgen, Boehringer Ingelheim, 10.13039/100004325AstraZeneca, 10.13039/100004334Merck, 10.13039/100004319Pfizer, 10.13039/100020323Aegerion, 10.13039/100004336Novartis, 10.13039/100004339Sanofi, Takeda, Novo Nordisk, and Bayer; and Honoraria from 10.13039/100002429Amgen, Boehringer Ingelheim, 10.13039/100004334Merck, 10.13039/100004319Pfizer, Aegerion, 10.13039/100004336Novartis, 10.13039/100004339Sanofi, Takeda, Novo Nordisk, and 10.13039/100004326Bayer.

All other authors have reported that they have no relationships relevant to the contents of this paper to disclose.
