# Supplementary material for: Geographic variation in surgery rates among older patients with early (ER positive HER2 negative) breast cancer: Influence of cardiovascular disease and comorbidities: A national registry dataset analysis
Source: Eur J Surg Oncol. 2025 Dec;51(12):None. doi: 10.1016/j.ejso.2025.110432 (PMC12673996; doi:10.1016/j.ejso.2025.110432)
Supplement: Multimedia component 1 [file mmc1.docx]

**3.1 Appendices**

**Supplementary Table A: Eligibility criteria for the analysis**

| **Inclusion criteria** | **Exclusion criteria** |
| --- | --- |
| - Cancer diagnosis in the period 2013-2018 - Cancer type and stage: - Stage I-III breast cancer (ICD-10: C50) - Lived in England - Finalised cancer registration | - Age <18 years or age >100 years at diagnosis - Missing NHS number - All behaviour codes except malignant - Missing mortality status - Death/censoring date before diagnosis date - Carcinoid morphology - Stages 3B or 4 at diagnosis - Missing disease stage at diagnosis - Diagnosis from death certificate only - Men diagnosed with breast cancer - Duplicates |

ICD-10: International Statistical Classification of Diseases and Related Health Problems-10

| **Supplementary Table B: SSISS Protocol: OPCS operating procedure codes**  **OPCS-4.8 to OPCS-4.9 Breast Surgery** | |
| --- | --- |
| B27 Total excision of breast (Clean) Note: Use a supplementary code for removal of lymph node (T85-T87) B27.1 Total mastectomy and excision of both pectoral muscles and part of chest wall B27.2 Total mastectomy and excision of both pectoral muscles NEC B27.3 Total mastectomy and excision of pectoralis minor muscle B27.4 Total mastectomy NEC Includes: Simple mastectomy B27.5 Subcutaneous mastectomy Includes: Nipple sparing mastectomy B27.6 Skin sparing mastectomy B27.8 Other specified B27.9 Unspecified Includes: Mastectomy NEC B28 Other excision of breast (Clean) Note: Use a supplementary code for removal of lymph node (T85-T87) B28.1 Quadrantectomy of breast B28.2 Partial excision of breast NEC Includes: Wedge excision of breast NEC Wide excision of breast NEC B28.3 Excision of lesion of breast NEC Includes: Lumpectomy of breast NEC B28.4 Re-excision of breast margins B28.6 Excision of accessory breast tissue B28.8 Other specified B28.9 Unspecified Breast reconstruction, mammoplasty, excision of lesion, tissue or breast including radical, modified, or quadrant resection, lumpectomy, incisional biopsy  B29 Reconstruction of breast (Clean) *Excludes: Reconstruction of breast using flap of skin of buttock (B38) *Reconstruction of breast using abdominal flap (B39) Note: Use a supplementary code for insertion of prosthesis for breast (B30.1) or skin expander (S48) Use a supplementary code for attention to skin expander (S49) B29.1 Reconstruction of breast using myocutaneous flap of latissimus dorsi muscle B29.2 Reconstruction of breast using local flap of skin NEC B29.3 Reconstruction of breast using flap of skin of abdomen NEC B29.4 Reconstruction of breast using distant flap of skin NEC B29.5 Revision of reconstruction of breast B29.8 Other specified B29.9 Unspecified B30 Prosthesis for breast (Clean) | Excludes: Augmentation mammoplasty (B31.2) B30.1 Insertion of prosthesis for breast B30.2 Revision of prosthesis for breast B30.3 Removal of prosthesis for breast B30.4 Renewal of prosthesis for breast B30.8 Other specified B30.9 Unspecified B31 Other plastic operations on breast (Clean) B31.1 Reduction mammoplasty B31.2 Augmentation mammoplasty B31.3 Mastopexy B31.4 Revision of mammoplasty B31.8 Other specified B31.9 Unspecified  B38 Reconstruction of breast using flap of skin of buttock (Clean) Note: Use a supplementary code for insertion of prosthesis for breast (B30.1) or skin expander (S48) Use a supplementary code for attention to skin expander (S49) B38.1 Reconstruction of breast using free superior gluteal artery perforator flap B38.2 Reconstruction of breast using free inferior gluteal artery perforator flap B38.8 Other specified B38.9 Unspecified B39 Reconstruction of breast using abdominal flap (Clean) Excludes: Reconstruction of breast using flap of skin of abdomen NEC (B29.3) Note: Use a supplementary code for insertion of prosthesis for breast (B30.1) or skin expander (S48) Use a supplementary code for attention to skin expander (S49) B39.1 Reconstruction of breast using free transverse rectus abdominis myocutaneous flap B39.2 Reconstruction of breast using pedicled transverse rectus abdominis myocutaneous flap B39.3 Reconstruction of breast using free deep inferior epigastric perforator flap B39.4 Reconstruction of breast using pedicle omental flap B39.5 Reconstruction of breast using free omental flap B39.8 Other specified B39.9 Unspecified O14 Other lymph node (Clean) O14.2 Sentinel lymph node T85 Block dissection of lymph nodes (Clean) T85.1 Block dissection of cervical lymph nodes T85.2 Block dissection of axillary lymph nodes T85.3 Block dissection of mediastinal lymph nodes T85.4 Block dissection of para-aortic lymph nodes T85.5 Block dissection of inguinal lymph nodes T85.6 Block dissection of pelvic lymph nodes T85.8 Other specified T85.9 Unspecified |

**Supplementary Table C: Definition of cardiovascular disease according to International Statistical Classification of Diseases and Related Health Problems (ICD)-10 codes**

| **ICD-10 code** | **Description** |
| --- | --- |
| **I05** | **Rheumatic mitral valve diseases** |
| **I05.0** | Mitral stenosis |
| **I05.1** | Rheumatic mitral insufficiency |
| **I05.2** | Mitral stenosis with insufficiency |
| **I05.8** | Other mitral valve diseases |
| **I05.9** | Mitral valve disease, unspecified |
| **I06** | **Rheumatic aortic valve diseases** |
| **I06.0** | Rheumatic aortic stenosis |
| **I06.1** | Rheumatic aortic insufficiency |
| **I06.2** | Rheumatic aortic stenosis with insufficiency |
| **I06.8** | Other rheumatic aortic valve diseases |
| **I06.9** | Rheumatic aortic valve disease, unspecified |
| **I08** | **Multiple valve diseases** |
| **I08.0** | Disorders of both mitral and aortic valves |
| **I08.1** | Disorders of both mitral and tricuspid valves |
| **I08.2** | Disorders of both aortic and tricuspid valves |
| **I08.3** | Combined disorders of mitral, aortic and tricuspid valves |
| **I08.8** | Other multiple valve diseases |
| **I08.9** | Multiple valve disease, unspecified |
| **I11** | **Hypertensive heart disease.** |
| **I11.0** | Hypertensive heart disease with (congestive) heart failure |
| **I13** | **Hypertensive heart and chronic kidney disease.** |
| **I13.0** | Hypertensive heart and renal disease with (congestive) heart failure |
| **I13.2** | Hypertensive heart and renal disease with both (congestive) heart failure and renal failure |
| **I20** | **Angina pectoris** |
| **I20.0** | Unstable angina |
| **I20.8** | Other forms of angina pectoris |
| **I20.9** | Angina pectoris, unspecified |
| **I21** | **Acute myocardial infarction** |
| **I21.0** | Acute transmural myocardial infarction of anterior wall |
| **I21.1** | Acute transmural myocardial infarction of inferior wall |
| **I21.2** | Acute transmural myocardial infarction of other sites |
| **I21.3** | Acute transmural myocardial infarction of unspecified site |
| **I21.4** | Acute subendocardial myocardial infarction |
| **I21.9** | Acute myocardial infarction, unspecified |
| **I22** | **Subsequent myocardial infarction** |
| **I22.0** | Subsequent myocardial infarction of anterior wall |
| **I22.1** | Subsequent myocardial infarction of inferior wall |
| **I22.8** | Other forms of acute ischaemic heart disease |
| **I22.9** | Acute ischaemic heart disease, unspecified |
| **I23** | **Certain current complications following ST elevation (STEMI) and non-ST elevation (NSTEMI) myocardial infarction (within the 28 day period)** |
| **I23.0** | Haemopericardium as current complication following acute myocardial infarction |
| **I23.1** | Atrial septal defect as current complication following acute myocardial infarction |
| **I23.2** | Ventricular septal defect as current complication following acute MI |
| **I23.3** | Rupture of cardiac wall without haemopericardium as current complication following acute myocardial infarction |
| **I23.4** | Rupture of chordae tendineae as current complication following acute myocardial infarction |
| **I23.5** | Rupture of papillary muscle as current complication following acute myocardial infarction |
| **I23.6** | Thrombosis of atrium, auricular appendage and ventricle as current complications following acute MI |
| **I23.8** | Other current complications following acute myocardial infarction |
| **I24** | **Other acute ischaemic heart diseases** |
| **I24.8** | Other forms of acute ischaemic heart disease |
| **I24.9** | Acute ischaemic heart disease, unspecified |
| **I25** | **Chronic ischaemic heart disease** |
| **I25.0** | Atherosclerotic cardiovascular disease, so described |
| **I25.1** | Atherosclerotic heart disease |
| **I25.5** | Ischaemic cardiomyopathy |
| **I25.8** | Other forms of chronic ischaemic heart disease |
| **I25.9** | Chronic ischaemic heart disease, unspecified |
| **I27** | **Other pulmonary heart diseases** |
| **I27.9** | Pulmonary heart disease, unspecified |
| **I34** | **Nonrheumatic mitral valve disorders** |
| **I34.0** | Mitral (valve) insufficiency |
| **I34.1** | Mitral (valve) prolapse |
| **I34.2** | Nonrheumatic mitral (valve) stenosis |
| **I34.8** | Other nonrheumatic mitral valve disorders |
| **I34.9** | Nonrheumatic mitral valve disorder, unspecified |
| **I35** | **Nonrheumatic aortic valve disorders** |
| **I35.0** | Aortic (valve) stenosis |
| **I35.1** | Aortic (valve) insufficiency |
| **I35.2** | Aortic (valve) stenosis with insufficiency |
| **I35.8** | Other aortic valve disorders |
| **I35.9** | Aortic valve disorder, unspecified |
| **I42** | **Cardiomyopathy** |
| **I42.0** | Dilated cardiomyopathy |
| **I42.5** | Other restrictive cardiomyopathy |
| **I42.6** | Alcoholic cardiomyopathy |
| **I42.7** | Cardiomyopathy due to drugs and other external agents |
| **I42.9** | Cardiomyopathy, unspecified |
| **I43** | **Cardiomyopathy in diseases classified elsewhere** |
| **I43.1** | Cardiomyopathy in metabolic diseases |
| **I43.8** | Cardiomyopathy in other diseases classified elsewhere |
| **I50** | **Heart failure** |
| **I50.0** | Congestive heart failure |
| **I50.1** | Left ventricular failure |
| **I50.9** | Heart failure, unspecified |
| **I60** | **Subarachnoid haemorrhage** |
| **I60.0** | Subarachnoid haemorrhage from carotid siphon and bifurcation |
| **I60.1** | Subarachnoid haemorrhage from middle cerebral artery |
| **I60.2** | Subarachnoid haemorrhage from anterior communicating artery |
| **I60.3** | Subarachnoid haemorrhage from posterior communicating artery |
| **I60.4** | Subarachnoid haemorrhage from basilar artery |
| **I60.5** | Subarachnoid haemorrhage from vertebral artery |
| **I60.6** | Subarachnoid haemorrhage from other intracranial arteries |
| **I60.7** | Subarachnoid haemorrhage from intracranial artery, unspecified |
| **I60.8** | Other subarachnoid haemorrhage |
| **I61** | **Intracerebral haemorrhage** |
| **I61.0** | Intracerebral haemorrhage in hemisphere, subcortical |
| **I61.1** | Intracerebral haemorrhage in hemisphere, cortical |
| **I61.2** | Intracerebral haemorrhage in hemisphere, unspecified |
| **I61.4** | Intracerebral haemorrhage in cerebellum |
| **I61.6** | Intracerebral haemorrhage, multiple localized |
| **I61.9** | Intracerebral haemorrhage, unspecified |
| **I62** | **Other nontraumatic intracranial haemorrhage** |
| **I62.1** | Nontraumatic extradural haemorrhage |
| **I62.9** | Intracranial haemorrhage (nontraumatic), unspecified |
| **I63** | **Cerebral infarction** |
| **I63.0** | Cerebral infarction due to thrombosis of precerebral arteries |
| **I63.1** | Cerebral infarction due to embolism of precerebral arteries |
| **I63.2** | Cerebral infarction due to unspecified occlusion or stenosis of precerebral arteries |
| **I63.3** | Cerebral infarction due to thrombosis of cerebral arteries |
| **I63.4** | Cerebral infarction due to embolism of cerebral arteries |
| **I63.5** | Cerebral infarction due to unspecified occlusion or stenosis of cerebral arteries |
| **I63.6** | Cerebral infarction due to cerebral venous thrombosis, nonpyogenic |
| **I63.8** | Other cerebral infarction |
| **I63.9** | Cerebral infarction, unspecified |
| **I64** | **Stroke, not specified as haemorrhage or infarction** |
| **I65** | **Occlusion and stenosis of precerebral arteries, not resulting in cerebral infarction.** |
| **I65.0** | Occlusion and stenosis of vertebral artery |
| **I65.1** | Occlusion and stenosis of basilar artery |
| **I65.2** | Occlusion and stenosis of carotid artery |
| **I65.3** | Occlusion and stenosis of multiple and bilateral precerebral arteries |
| **I65.8** | Occlusion and stenosis of other precerebral artery |
| **I65.9** | Occlusion and stenosis of unspecified precerebral artery |
| **I66** | **Occlusion and stenosis of cerebral arteries, not resulting in cerebral infarction.** |
| **I66.0** | Occlusion and stenosis of middle cerebral artery |
| **I66.1** | Occlusion and stenosis of anterior cerebral artery |
| **I66.2** | Occlusion and stenosis of posterior cerebral artery |
| **I66.3** | Occlusion and stenosis of cerebellar arteries |
| **I66.4** | Occlusion and stenosis of multiple and bilateral cerebral arteries |
| **I66.8** | Occlusion and stenosis of other cerebral artery |
| **I66.9** | Occlusion and stenosis of unspecified cerebral artery |
| **I67** | **Other cerebrovascular diseases** |
| **I67.0** | Dissection of cerebral arteries, nonruptured |
| **I67.1** | Cerebral aneurysm, nonruptured |
| **I67.2** | Cerebral atherosclerosis |
| **I67.8** | Other specified cerebrovascular diseases |
| **I67.9** | Cerebrovascular disease, unspecified |
| **I69** | **Sequelae of cerebrovascular disease** |
| **I69.0** | Sequelae of subarachnoid haemorrhage |
| **I69.3** | Sequelae of cerebral infarction |
| **I69.4** | Sequelae of stroke, not specified as haemorrhage or infarction |
| **I69.8** | Sequelae of other and unspecified cerebrovascular diseases |
| **I70** | **Atherosclerosis** |
| **I70.0** | Atherosclerosis of aorta |
| **I70.1** | Atherosclerosis of renal artery |
| **I70.2** | Atherosclerosis of arteries of the extremities |
| **I70.8** | Atherosclerosis of other arteries |
| **I71** | **Aortic aneurysm and dissection** |
| **I71.0** | Dissection of aorta [any part] |
| **I71.1** | Thoracic aortic aneurysm, ruptured |
| **I71.2** | Thoracic aortic aneurysm, without mention of rupture |
| **I71.3** | Abdominal aortic aneurysm, ruptured |
| **I71.4** | Abdominal aortic aneurysm, without mention of rupture |
| **I71.5** | Thoracoabdominal aortic aneurysm, ruptured |
| **I71.6** | Thoracoabdominal aortic aneurysm, without mention of rupture |
| **I71.8** | Aortic aneurysm of unspecified site, ruptured |
| **I71.9** | Aortic aneurysm of unspecified site, without mention of rupture |
| **I72** | **Other aneurysm** |
| **I72.0** | Aneurysm of carotid artery |
| **I72.1** | Aneurysm of artery of upper extremity |
| **I72.2** | Aneurysm of renal artery |
| **I72.3** | Aneurysm of iliac artery |
| **I72.5** | Aneurysm of artery of other precerebral arteries |
| **I72.6** | Aneurysm and dissection of vertebral artery |
| **I72.8** | Aneurysm of other specified arteries |
| **I72.9** | Aneurysm of unspecified site |
| **I73** | **Other peripheral vascular diseases** |
| **I73.8** | Other specified peripheral vascular diseases |
| **I73.9** | Peripheral vascular disease, unspecified |
| **I74** | **Arterial embolism and thrombosis** |
| **I74.0** | Embolism and thrombosis of abdominal aorta |
| **I74.1** | Embolism and thrombosis of other and unspecified parts of aorta |
| **I74.2** | Embolism and thrombosis of arteries of the upper extremities |
| **I74.3** | Embolism and thrombosis of arteries of the lower extremities |
| **I74.4** | Embolism and thrombosis of arteries of extremities, unspecified |
| **I74.5** | Embolism and thrombosis of iliac artery |
| **I74.8** | Embolism and thrombosis of other arteries |
| **I74.9** | Embolism and thrombosis of unspecified artery |
| **I77** | **Other disorders of arteries and arterioles** |
| **I77.3** | Arterial fibromuscular dysplasia |
| **I77.4** | Coeliac artery compression syndrome |
| **I79** | **Disorders of arteries, arterioles and capillaries in diseases classified elsewhere** |
| **I79.0** | Aneurysm of aorta in diseases classified elsewhere |
| **I79.2** | Peripheral angiopathy in diseases classified elsewhere |

ICD-10: International Statistical Classification of Diseases and Related Health Problems-10

**Supplementary Table D: Classifications of cardiovascular disease admission codes identified in Hospital Episode Statistics) prior to cancer diagnosis.**

| **Source** | **Phenotype** | **ICD-10 codes** | **Total (N=102,604) n (%)** |
| --- | --- | --- | --- |
| **Welch et al. (2020)^a^** | **Cerebrovascular** | I60 - I69 (excluding I60.9, I61.3, I61.5, I61.8, I61.8, I61.9, I62.0, I67.3, I67.4, I67.6, I67.7, I68.0, I68.2, I69.1 and I69.2) | 20,366 (19.9) |
|  | **Stroke (Cerebrovascular subgroup)** | I61 - I64 (excluding I60.9, I61.3, I61.5, I61.8 and I62.0), I69.0, I69.3 and I69.4 | 8,602 (8.4) |
|  | **Congestive cardiac failure** | I11.0, I13.0, I13.2, I42.0, I42.5, I42.6, I42.7, I42.9, I43.1, I43.8, I50.0, I50.1 and I50.9 | 21,462 (20.9) |
|  | **Ischaemic heart disease** | I20.0 - I25 (excluding I20.1 and I24.1) | 64,620 (63.0) |
|  | **Acute myocardial infarction (Ischaemic heart disease subgroup)** | I21 - I23 | 10,401 (10.1) |
|  | **Peripheral artery disease** | I70 - I74 (excluding I70.9, I72.4, I73.0 and I73.1),  I77.3, I77.4, I79.0, I79.2 and I84.6 | 20,911 (20.4) |
|  | **Valvular heart disease** | I105, I106, I108, I27.9, I34, I35, I31.1, I135.2, I35.5 and I35.9 | 17,770 (17.3) |

^a^ Welch CA, Sweeting MJ, Lambert PC, Rutherford MJ, Jack HR, West D, Adlam D, Peake M. Impact on survival of modelling increased surgical resection rates in patients with non-small-cell lung cancer and cardiovascular comorbidities: a VICORI study. Br J Cancer 123, 471–479 (2020). <https://doi.org/10.1038/s41416-020-0869-8>

**Supplementary Table E: Calculation of the Charlson Comorbidity Index with and without inclusion of cardiovascular diseases**

| **Charlson Group** | **Description** | **Charlson Score** | **Notes** |
| --- | --- | --- | --- |
| 1 | Acute myocardial infarction | 1 | Excluded from CVD-free comorbidity score |
| 2 | Congestive heart failure | 1 |  |
| 3 | Peripheral vascular disease | 1 |  |
| 4 | Cerebral vascular accident | 1 |  |
| 5 | Dementia | 1 | - |
| 6 | Pulmonary disease | 1 | - |
| 7 | Connective tissue disorder | 1 | - |
| 8 | Peptic ulcer | 1 | - |
| 9 | Diabetes | 1 | Only highest score is counted |
| 10 | Diabetes complications | 2 |  |
| 11 | Paraplegia | 2 | - |
| 12 | Renal disease | 2 | - |
| 13 | Cancer | 2 | Derived from cancer registry data rather than HES data |
| 14 | Metastatic cancer | N/A |  |
| 15 | Liver disease | 1 | Only highest score is counted |
| 16 | Severe liver disease | 3 |  |
| 17 | HIV | 6 | - |
